# Investigation of Stent Prototypes for the Eustachian Tube in Human Donor Bodies

**DOI:** 10.3390/bioengineering10060743

**Published:** 2023-06-20

**Authors:** Lena Rosenbusch, Robert Schuon, Tamara Wilfling, Philipp Krüger, Kerstin Lebahn, Samuel John, Olga Sahmel, Niels Grabow, Marko Schulze, Andreas Wree, Klaus-Peter Schmitz, Tobias Stein, Thomas Lenarz, Gerrit Paasche

**Affiliations:** 1 Department of Otorhinolaryngology, Hannover Medical School, Carl-Neuberg-Str. 1, 30625 Hannover, Germany; lena.rosenbusch@web.de (L.R.); schuon.robert@mh-hannover.de (R.S.); tamara.wilfling@yahoo.com (T.W.); lenarz.thomas@mh-hannover.de (T.L.); 2 Bess Pro GmbH, Gustav-Krone-Str. 7, 14167 Berlin, Germany; p.krueger@besspro.eu (P.K.); t.stein@bessgroup.com (T.S.); 3 Institute for Biomedical Engineering, Rostock University Medical Center, 18119 Rostock, Germany; kerstin.lebahn@uni-rostock.de (K.L.); olga.sahmel@uni-rostock.de (O.S.); niels.grabow@uni-rostock.de (N.G.); klaus-peter.schmitz@uni-rostock.de (K.-P.S.); 4 HörSys GmbH, 30625 Hannover, Germany; john.samuel@hoersys.de; 5 Institute of Anatomy, Rostock University Medical Center, 18057 Rostock, Germany; marko.schulze@uni-bielefeld.de (M.S.); andreas.wree@med.uni-rostock.de (A.W.); 6 Cluster of Excellence Hearing4all, Hannover Medical School, Carl-Neuberg-Str. 1, 30625 Hannover, Germany

**Keywords:** eustachian tube, nitinol stent, polymeric stent, 3D reconstruction, histologic evaluation

## Abstract

Chronic otitis media is often connected to Eustachian tube dysfunction. As successful treatment cannot be guaranteed with the currently available options, the aim is to develop a stent for the Eustachian tube (ET). Over the course of this development, different prototypes were generated and tested in ex vivo experiments. Four different prototypes of an ET stent and one commercially available coronary stent were implanted in the ET of seven human donor bodies. The position of the stents was verified by cone beam CT. The implanted ETs were harvested, embedded in resin and ground at 200 µm steps. Resulting images of the single steps were used to generate 3D models. The 3D models were then evaluated regarding position of the stent in the ET, its diameters, amount of squeezing, orientation of the axes and other parameters. Virtual reconstruction of the implanted ET was successful in all cases and revealed one incorrect stent placement. The cross-section increased for all metal stents in direction from the isthmus towards the pharyngeal orifice of the ET. Depending on the individual design of the metal stents (open or closed design), the shape varied also between different positions along a single stent. In contrast, the cross-section area and shape remained constant along the polymeric prototype. With the current investigation, insight into the behavior of different prototypes of ET stents was gained, which can help in defining the specifications for the intended ET stent.

## 1. Introduction

The Eustachian tube (ET) is a system that connects the middle ear spaces with the nasopharynx. Its tasks are many. Pressure equalization, middle ear ventilation, protection against sound and ascending germs, and drainage of middle ear secretions are only the most important ones mentioned [1].

Anatomic and physiologic knowledge is essential to understanding how Eustachian tube dysfunction (ETD) occurs. The ET consists of a cartilaginous part anteromedially, which takes about two thirds of its entire length, and of a bony part posterolaterally. The flexible cartilaginous part is attached to the musculature, which enables opening of the tube and thus ventilation of the middle ear by contraction in a complex synergism [2]. Dysfunction of the ET is—by strict definition—a failure of all the above-mentioned functions [3]. However, since ETD is primarily understood as a symptom complex of signs of pressure regulation dysfunction in the middle ear, ventilation dysfunction is the primary problem in ETD. Patients with ETD suffer from feelings of pressure, pain, discomfort, “popping”, and “aural fullness” [3].

A distinction is made between obstructive, dynamic, or combined disorders, the former of which may be mechanical or functional in origin. Obstructive disorders may be extrinsic or intrinsic, congenital or acquired and are often associated with mucosal edema or hyperplasia of various etiologies. Dynamic problems originate from dysfunction of the involved muscles tensor veli palatini (TVP) and/ or levator veli palatini (LVP) [4]. Consequences of a chronically disturbed tube ventilation can be negative pressure in the middle ear, chronic mesotympanic, and epitympanal otitis media (COM), adhesive processes of the middle ear and tympanosclerosis [5].

The idea of re-opening an obstructive tube from the outside is by no means an idea of modern times. The first developments of tubal catheterization can be traced back to the 18th century with documented studies, in which Jonathan Wathen (1728–1808) recorded pictorially and in writing how an exact placement of a catheter in the ET was performed [6].

In the last century, conservative approaches, such as Valsalva maneuver to equalize the pressure in the middle ear, nasal irrigation with saline solution or application of decongestants, antihistamines or corticosteroids, were introduced [5]. Additionally, more and more mechanical interventions have gained attention. These include laser tuboplasty, in which hypertrophic mucosa is vaporized [7]. In addition, Balloon Eustachian Tuboplasty (BET) emerged. Here, a transnasal applicator is placed in the tubal bulge, then the balloon catheter is advanced into the ET and dilated [8,9]. This is typically performed under additional endoscopic control [10].

Despite the promising results described [8,10], none of the interventions for the treatment of ETD can be recommended at this time due to lack of general effectiveness [11]. In a retrospective study after BET, it was shown that less than half of the patients experienced a subjective positive effect [12]. Accurate selection of the patient population should be undertaken to improve the success rate [12]. Another work showed that BET is safe, and the more superior option compared with conservative management alone [13].

Stent implantation in the ET is another intervention that is subject of current research to treat ETD. Implantation and tolerability of stents have already been successfully demonstrated in sheep and pig models [14,15]. These experiments were performed with conventional coronary cobalt chromium (CoCr) stents. Additionally, one group has shown that intraoperative stent insertion in a human patient is technically feasible [16].

Implanting a stent into the human ET provides chances for a better treatment of patients, but several challenges have to be addressed during development. A stent shall be placed in the cartilaginous part of the ET and shall keep the ET lumen open. It can be expected that connective tissue is formed around the struts [14,15] that reduces the lumen again, and the mucosa might get damaged during insertion and/or expansion of the stents. Furthermore, creating a patulous tube has to be avoided, the stent should facilitate the natural function of the ET. Therefore, length, diameter, position in the ET, shape, choice of material, and other properties will have an influence on the results with stents in patients and have to be addressed during development.

An extensive study on the dimensions and position of the human ET based on cone beam computed tomography (CBCT) scans provided valid information for a possible treatment of the human ET with stents [17]. This enables the development of stents especially for application in the human ET. The decisive factor here might be how long a support of the ET by a stent is needed. This determines the material to be used as different materials meet different requirements.

If a permanent support is needed, CoCr could be used, as well as Nitinol. Force is needed to open CoCr stents and these are typically used in blood vessels, whereas Nitinol stents always want to keep the shape in which they were produced. Nitinol stents are currently used in numerous fields of application, such as intraluminal in vessels in peripheral artery disease [18], esophageal in the gastrointestinal tract [19], or intracranial [20]. For use in the ET with its special requirements such as an intermittent dynamic opening, a CoCr stent might not be the best option. 

If only short-term support is wanted, a degradable stent would be preferred. For example, the use of biodegradable polymeric stents in percutaneous coronary interventions is being investigated [21]. Alternatively, magnesium-based stents have promising properties due to their biocompatibility and biodegradability [22]. Both types of stents are already used in human application [23,24]. However, iron-based stents are also being explored as biodegradable stents [25].

Within the current study, the behavior of different stent prototypes made from Nitinol or polymeric materials was investigated after implantation into the ET of human body donors. The stent prototypes were compared to an established CoCr coronary stent. An evaluation of the insertion characteristics of the stents or the best position of the stent in the ET was not the purpose of this study. This has to be investigated in further studies.

## 2. Materials and Methods

### 2.1. Ethics Approval

The study was performed on five (two implanted on both sides) donor cadavers (Caucasian; two males and three females; age: 72–88 years) who had donated their bodies for education or research purposes to the Institute of Anatomy, Rostock University Medical Center, Germany. The Ethics Committee of the University of Rostock approved the use of the bodies for research purposes under number A 2016 0083. The local Ethics Committee of Hannover Medical School approved the analysis of the stent prototypes in the ET under number 8515_BO_K_2019. 

### 2.2. Materials

Three different prototypes of a Nitinol (NiTi) stent (bess pro GmbH, Berlin, Germany) were used in the study. Two of them had a closed design, and one an open design. All had a nominal diameter of 5 mm and a length of 15 mm. These stents were fixed on a tool and released after insertion in the human ET. In addition, a prototype stent made from a poly-L-lactic acid (PLLA)-based polymeric blend (IBMT, Rostock University Medical Center, Rostock, Germany) was used. This stent had a length of 20 mm and was crimped on a conventional balloon catheter for insertion in the ET. In addition, a conventional Pro-Kinetic Energy cobalt chromium (CoCr) coronary stent (Biotronik AG, Bülach, Switzerland) was used to implant one ET. An overview of the stents and their distribution among the donors is provided in Table 1.

### 2.3. Insertion

The insertion of the different stent prototypes into the ET was performed by experienced ENT surgeons through the nose via a T-Tube introducer (Spiggle+Theis Medizintechnik GmbH, Overath, Germany). The Nitinol stents were self-expanding. The polymer as well as the CoCr stent were inflated by a balloon catheter (12 bar for 1 min) and thus opened.

The insertion was performed under endoscopic control from the contralateral side via a 30° optic connected to an AIDA system (Karl Storz SE and Co. KG, Tuttlingen, Germany).

### 2.4. Preparation, Fixation, Embedding of the Samples

The donors were pre-fixed in 4% formaldehyde solution. Preparation of the temporal bones from the skulls was performed at the Department of Anatomy at the Rostock University Medical Center. Before further preparation for histologic evaluation of the ETs, samples had to be trimmed by use of a bone saw (Bizerba SE and Co. KG, Balingen, Germany) to bring them to a suitable size for the cylindrical casting mold (diameter: 35 mm, height: 70 mm). Once the final shape and size were achieved, the samples were rinsed with phosphate-buffered solution for one hour as an intermediate step to wash the formaldehyde solution out. An ascending alcohol series was applied to dehydrate the samples. In this process, the samples were stored in 50, 70, 90, and 100% ethanol for one week each, and in a last step, the ethanol was replaced by 100% methanol for another three days. The samples were then embedded in epoxy under addition of UVO™ color pigment/1 white (Smooth-On, Inc., Macungie, PN, USA).

After embedding in epoxy resin under vacuum degassing, the test specimens were cured in the mold. The mold had two characteristic grooves, which were crucial for the processing of the image material.

### 2.5. Cone Beam CT Scans

The cured cylindrical preparations were radiographed using cone beam computed tomography (CBCT) (3D Accuitomo XYZ Slice View Tomograph, J. MORITA MFG. Corp., Kyoto, Japan), FOV 80 × 80. The images were acquired using the following settings: 30.8 s exposure time, CTDI vol: 11.6 mGy, DLP: 93.0 mGy × cm, Mode: CT, 360° scan, HiFi imaging.

### 2.6. Series Grinding and Documentation

Wet grinding with SiC paper of grit size P400 or P1200 was performed by two-stage removal of the exposed plane using an AutoMet250 Pro grinding machine (BUEHLER AG, Uzwil, Switzerland). The paper rotated at 500 min^−1^, the sample holder at 60 min^−1^ in the same direction, and the grinding pressure force was set to 45 N. The set grinding depths were 0.90 mm (P400 grit) and 0.10 mm (P1200 SK). Thus, a total of 1 mm of stock removal per plane was achieved. The depth was refined to 200 µm when the stent was reached. Here, 0.10 mm were removed by each of the two paper grits, i.e., 0.2 mm per plane were removed. This approach was applied when grinding the first series of samples, i.e., samples NiTi 1b, NiTi 3b, Polymer and CoCr. In the second series with samples NiTi 1a, NiTi 2 and NiTi 3a, a uniform grinding depth of 0.20 mm was applied.

The different grinding planes were documented using a VHX Digital Microscope VH-Z20 UR with universal zoom lens (Keyence Ltd., Osaka, Japan) at a magnification of 20× by using image stitching. White balancing was performed before starting to collect the microscopic images.

### 2.7. Data Processing

The raw images were aligned by aligning the grooves generated by the casting mold that were visible in each image. This was performed via a custom-made software (HörSys GmbH, Hannover, Germany). Such, a stack of images was created that was then loaded into 3D Slicer (version 4.11.0) software (https://www.slicer.org, accessed on 1 December 2022) [26] to generate a 3D dataset of the stent and its surrounding area. For this reconstruction, only the regions of the stents were used that were ground at 200 µm distance between the grinding planes. To detect possible inaccuracies of the grinding, the histologic 3D model was fused with the CBCT image. 

**Figure 1:**
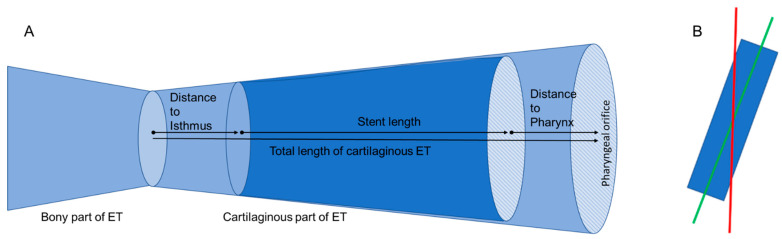
(**A**) Schematic drawing of the ET and the measured lengths and distances. (**B**) Axis shift of the grinding plane. The red line symbolizes the axis of the epoxy block (grinding planes), the green line the axis of the stent (virtual plane for evaluation of the stent).

In the fused images, measurements such as the distance of the stent from the isthmus region of the ET were made with the Ruler Tool in 3D Slicer to determine the position of the stent within the tube (Figure 1A). The lengths of the ET itself and the length of the stents were determined. Furthermore, the Segmentation Tool was used to measure the open area within the stent in the ET in cross-section by means of automatic contrasting. The area of the stent can be approximated with the help of an ellipse. This approach was used to validate the approximation by an ellipse against the automatically determined area of the stent.

Since the stent was not oriented parallel to the axis of the cylindrical epoxy block, measurement inaccuracies were suspected. The main axis of the 3D model in 3D Slicer was therefore adjusted to the main axis of the stent (compare Figure 1B).

**Figure 2:**
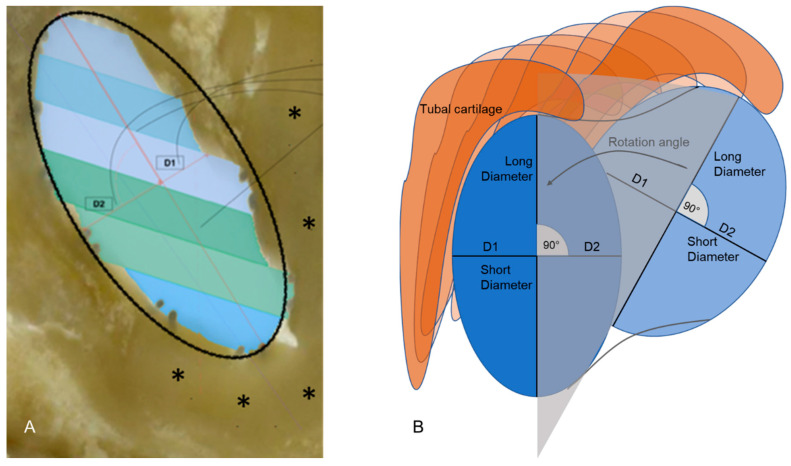
(**A**) Representation of the approximated ellipse, the long and short diameter, and the division of the short diameter into D1 and D2. The tubal cartilage is marked by asterisks. The different colors in the lumen of the ET indicate the different histologic sections contributing to this virtual slice through the 3D model. (**B**) Schematic presentation of a section through stent and ET perpendicular to the axis of the stent. Different measured diameters and lengths, and a rotation of the long axis following the stent in the ET are indicated.

For all further measurements, the cross-sections perpendicular to the axis of the stents were used (Figure 2). The longest diameters of the stents were determined and the diameters perpendicular to it (short diameters), dividing the longest diameter into two equal halves. By changing the main axis, the Segmentation Tool for automated determination of an area could no longer be used. The cross-section area of the stent was now approximated by calculating the elliptical area from the two measured diameters (longest and perpendicular to it) (Figure 2A). These measurements were performed over the entire length of the stent. As both parts of the short diameter were not always of equal length (compare also Figure 2A), the lengths of both parts were determined additionally. With the main axis corrected, the rotation of the longest diameter within the tube could be followed from one image plane to the next from the pharyngeal ET ostium in the direction of the isthmus (Figure 2B). To compare different stent prototypes, it was concentrated on a region of the ET in which all stents were placed. This was performed to minimize the influence of differences in tube anatomy.

## 3. Results

**Figure 3:**
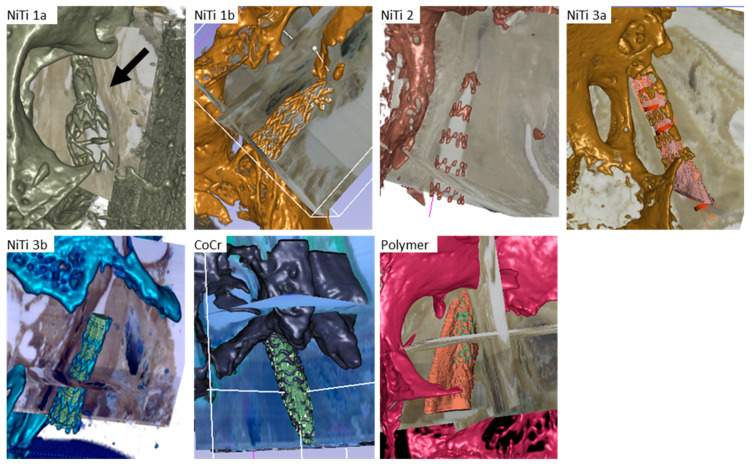
Merged images of CBCT scans and histology. The metal stents could be visualized in the CBCT scans. The Polymer stent had to be displayed by the Segmentation Tool of the histologic model where the light red color shows the lumen of the ET, the shimmering green depicts the stent.

All stents were inserted into the ET without difficulty. For all stents, the histologic 3D model was fused with the CBCT images (overview provided in Figure 3). This resulted in a detected inaccuracy of the grinding process of 84 µm per plane on average as determined for stent NiTi 3a. The distances between the different planes were adjusted accordingly in the 3D model in all planes and all samples before performing any measurements. However, one of the stents (NiTi 1b) was found to be incorrectly placed in CBCT and histology. It perforated the tubal wall in caudal direction. This stent was not included in further data evaluation. The six remaining stents were all positioned in the respective ETs without any detected damage, even though not all stents seem to be completely unfolded over the entire length (compare especially NiTi 1a).

**Figure 4:**
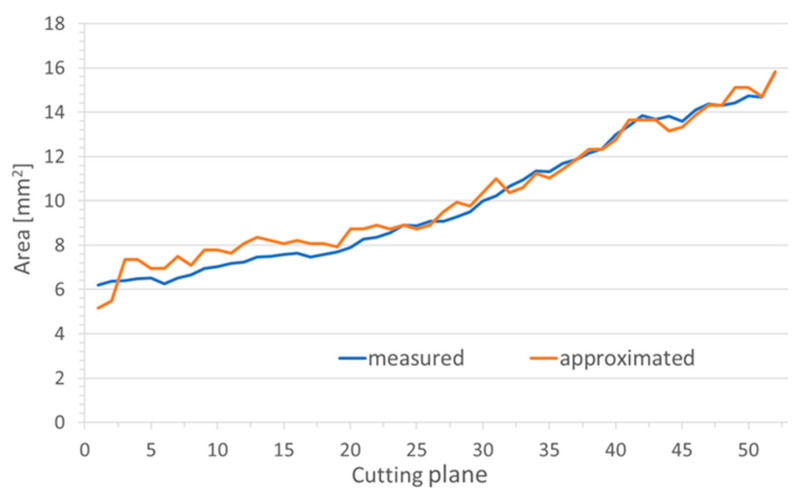
Comparison of the stent area of NiTi 3b as measured by the software and the area as approximated by an ellipse.

In the virtual plane perpendicular to the stent axis, the area of the stent could not directly be measured but had to be approximated by an ellipse. Therefore, in the grinding planes, areas were also approximated by an ellipse and compared to the directly measured areas (Figure 4). The difference between the measured and calculated cross-sectional areas of the lumen kept open by the stent was between 0.001 mm^2^ and 5.298 mm^2^, both measured for stent NiTi 1a.

On average, the calculated elliptical area was a little larger than the measured stent area. Only the polymer stent showed on average a 0.029 mm^2^ larger measured area than the calculated elliptical area. The largest mean deviation was found in the Nitinol stent NiTi 2 with 1.477 mm^2^, followed by the Nitinol stent NiTi 1a with 0.534 mm^2^ and CoCr 0.277 mm^2^. On average, the smallest deviations were found in stent NiTi 3b with 0.263 mm^2^ and stent NiTi 3a with 0.003 mm^2^.

All metal stents had nominal lengths of 15 mm. For two of the NiTi stents (NiTi 3b and NiTi 2), the measured lengths were 0.7 to 0.8 mm larger, whereas for the CoCr and NiTi 1a the measured lengths were only 13.2 and 13.1 mm, respectively (compare Table 2). The polymer stent was measured to be 19.9 mm long, which nearly matches its nominal length of 20 mm. The position of the stents inside the ET were highly variable. The polymer stent was inserted through the isthmus of the ET by 6.8 mm before inflation. The NiTi stent with the open design (NiTi 2) was not completely positioned in the ET but protruded into the pharyngeal space by about 3 mm (Table 2). Nevertheless, there remained a region of 7.2 mm of the cartilaginous part of the ET, which was covered by all stent prototypes. The average length of the cartilaginous part of the ET was 22.4 mm.

When looking at the area kept open by the stent in cross-section, the largest area was found to be 15.1 mm^2^ (NiTi 3b), and the smallest area was 1.5 mm^2^ (NiTi 1a) in a single plane. The maximum expected areas were 19.6 mm^2^ for NiTi stents, 7.1 mm^2^ for the CoCr stent and 9.6 mm^2^ for the polymer stent.

**Figure 5:**
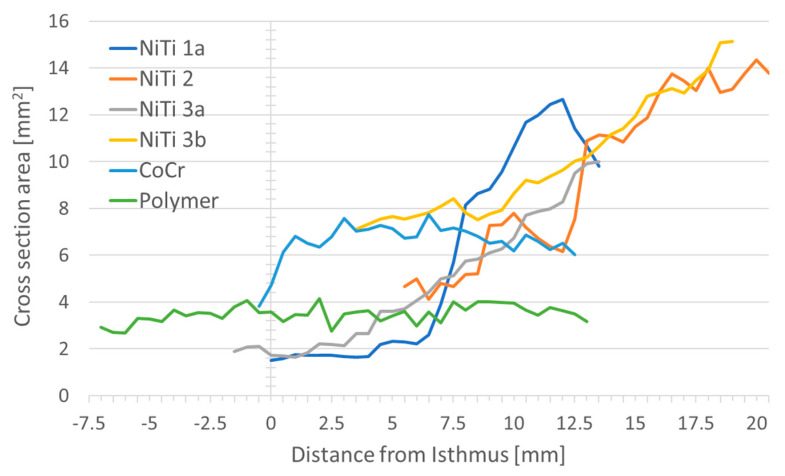
Position of the stent prototypes in the ET and cross-sectional area at the different positions. Zero on the x-axis refers to the isthmus for each ET. The position of the lines indicates the position of the stent in the ET. Positive values on the x-axis indicate the distance from the isthmus in the cartilaginous part of the ET, negative values indicate the distance from the isthmus in direction to the middle ear.

The average open area was largest for Nitinol stent NiTi 3b (10.0 mm^2^). The smallest open areas on average were found for the Nitinol stent NiTi 3a with 4.9 mm^2^ and the polymer stent (3.5 mm^2^). In general, the area in cross-section, and thus the lumen, kept open by the respective stents increased from the isthmus to the pharynx for all prototypes. The polymer stent was the only stent with hardly any change in area over the length of the stent (Figure 5).

There was approximately a doubling in the area from the isthmus to the pharynx in the NiTi 3b stent. About a threefold increase was seen in the remaining Nitinol stents (NiTi 1a, NiTi 2, NiTi 3a), whereas with CoCr a smaller area was found at the isthmus and the cross-sectional area was almost constant throughout the rest of the stent. A constant cross-sectional area was also found with the polymer stent. An overview of the stent area and the referring diameters at both ends of the stents is provided in Table 3. To facilitate visual imagination, please refer to Figure 1B and Figure 2B.

**Figure 6:**
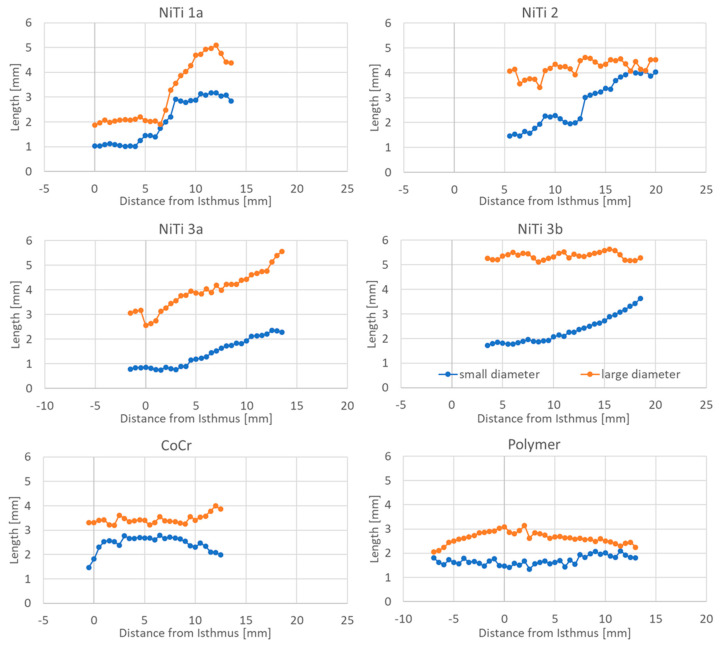
Large and small diameters of the stents in relation to the position of the stent in the ET. Zero on the x-axis refers to the isthmus for each ET. The larger the distance between both curves, the more flattened appeared the stent. In stent NiTi 1a, large and small diameters swapped at about 7 mm distance from the isthmus. For stent NiTi 2, a periodic decrease in the large diameter is obvious.

The long and short diameters of the cross-section of the stent provide an indication of the shape of the stent in the ET lumen. While an approximately round shape was observed for the polymer stent both in the pharyngeal region and at the end facing the middle ear, and for the stent NiTi 2 in the pharyngeal region (this stent protruded into the pharyngeal space), this was not seen for any other stent. Here, larger differences between short and long diameters were found, indicating a more elliptical shape of the stents. The shape of all stents varied over the entire lengths of the stents (Figure 6).

Insertion depth seemed to determine the shape of the stents. The closer a NiTi stent was positioned to the isthmus, the larger was the deviation from a round shape. Only when the position of the stent was close to the pharyngeal orifice, short and long diameters tended to converge. Additionally, the polymeric stent deviated from its round shape only in the isthmus region (Figure 6). The stent NiTi 1a presented in the isthmus region over a length of approximately 3 mm constant values for short and long diameters. Then, there was a caliber jump (compare also Figure 3), during which the short and long diameters swap.

**Figure 7:**
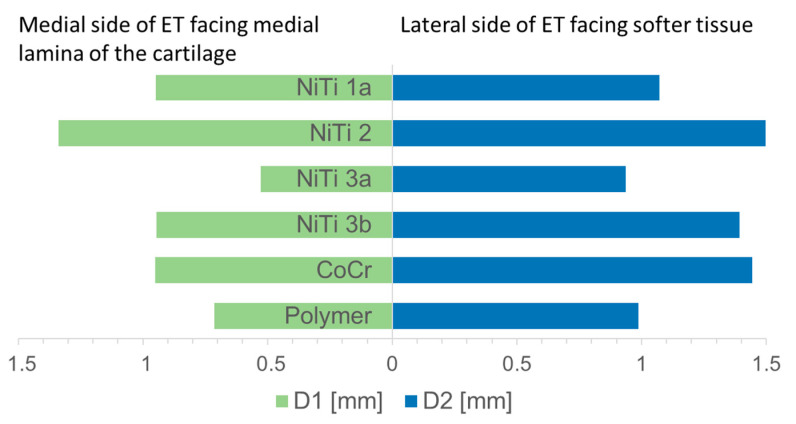
Comparison between both parts of the short diameter.

Per definition, the short diameter of the stent divides the longest diameter in two equal halves and is perpendicular to the longest diameter. Both parts of the short diameter as generated by the intersection with the longest diameter were not of equal length. The portion of the short diameter facing the tubal cartilage was in all stents smaller than the other portion (Figure 7).

**Figure 8:**
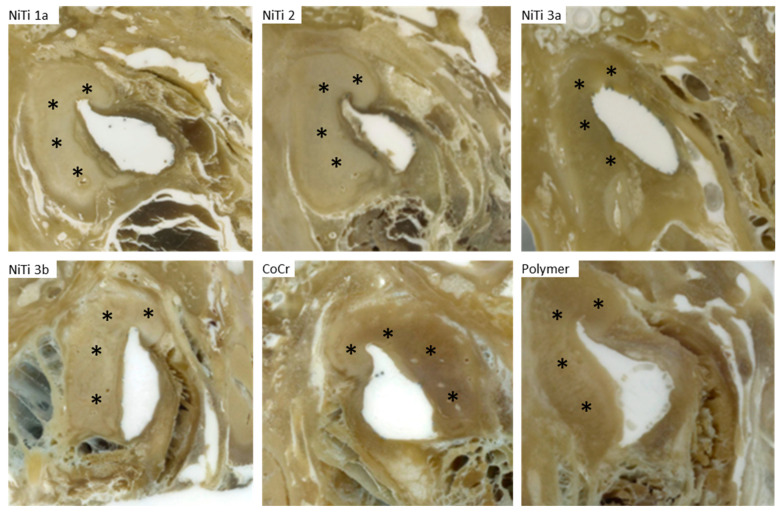
Histologic images of grinding planes at 6 mm distance to isthmus. The tubal cartilage is marked by asterisks. All NiTi stents appear more flattened than CoCr and polymer stents.

Due to the different positions of the stents in the ET, direct comparison between the different prototypes appeared to be challenging. Therefore, a position in the ET at 6 mm distance from the isthmus was chosen, where all stents were located (Figure 8).

At this position, the areas of the stent in the cross-section were between 4.0 mm^2^ and 7.7 mm^2^ (compare Table 4), with the smallest one referring to the polymeric stent followed by the NiTi stent with the open design (NiTi 2). The measured diameters strongly indicate an ellipsoidal shape for the Nitinol stents NiTi 3b, NiTi 2, and NiTi 3a, where the long diameter is more than twice as long as the short diameter (Table 4). Only stent NiTi 1a, the one that was not completely unfolded, and the cobalt-chromium and polymer stents show a more rounded shape.

**Figure 9:**
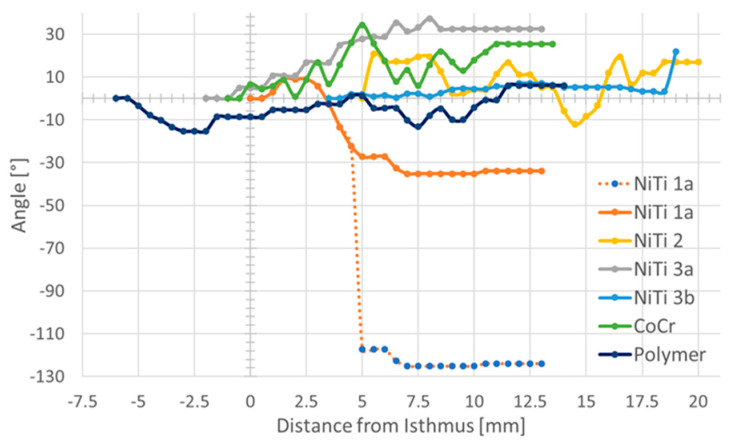
Rotation of the longest axis of the stents. The first cross-section of the stent at the side facing the middle ear was taken as origin and angles were then always measured in relation to this first image. Zero on the x-axis refers to the isthmus for each ET. For stent NiTi 1a, the measured values (blue dots) and the 90° corrected values (solid line) are provided. Note that in the region with the perceived negative rotation, this stent was not unfolded.

By following the longest axis of the stents through all image planes, it was observed that the orientation of the axis was not stable, but rotated slightly. All but one of the stents rotated laterally with the caudal edge of cartilage in the direction from the isthmus region to the nasopharynx. The only exception was again the stent in NiTi 1a. Here, the behavior was comparable to the other stents in the half that was completely unfolded, but when reaching the part where the long and short diameters also swapped, it became different, resulting in an overall perceived rotation in the opposite direction (Figure 9). 

## 4. Discussion

Stents might be a promising option to treat ETD. Implantation of stents in the ET was feasible and well tolerated, at least in sheep [14]. These earlier experiments were performed using standard CoCr coronary stents. The use of coronary stents must not be the ideal solution for the ET. In addition, patients might need chronic support for the ET or only temporary support. Therefore, the purpose of the current study was to investigate the first prototypes of a stent for the ET in human donor cadavers. The focus was on the behavior of the stents in the tissue in relation to the design and material of the stents.

While CoCr stents have been extensively studied and used in blood vessels [27], Nitinol stents are also used in a variety of parts of the gastrointestinal system [28], or in the trachea [29]. Especially, the use of biodegradable stents is currently the subject of further research although they are used in clinical practice [30].

To investigate the behavior of the stents, implanted ETs were ground and documented. As the accuracy of the distances achieved with this method can be influenced by different parameters such as asymmetry of grinding or different degrees of hardness of the preparations [31], data sets from CBCT and histology were superimposed. This approach has successfully been used in processing the ET of a black-face sheep to generate a 3D model of the ET [32]. Here, the samples were sawed, but the general problem remains the same. When fusing CBCT and histologic images based on bone structures and metal stents, histological data had to be adjusted. Differences between CBCT and histology can primarily be explained by the above-mentioned inaccuracies in the grinding process. In the CBCT scan, the expected error should be about twice the size of a voxel, in our case 0.08 mm. The correction of 84 µm per image plane was an average value for the temporal bone implanted with NiTi 3a, that was then applied to all planes and all samples. For a specific plane and the other samples, it could be under or overestimated. Therefore, this might also contribute to the differences in lengths found for the NiTi stents (all should have a length of 15 mm) as measurements were performed in the corrected histologic 3D model. This is supported by the fact that in samples NiTi 2 and NiTi 3b the length of the stents was overestimated (more material removed per plane), and these stents were positioned at larger distances from the isthmus resulting in more soft tissue and less bone in the sample. Therefore, all distances were provided with only one decimal place even though the measurement tools provided three decimal places. Furthermore, when expanding a stent, its length will reduce slightly. The amount of shrinkage will depend on the material and design of the stent. As different prototypes were used in the current study, shrinkage probably has influenced all stents differently to a minor degree.

Despite grinding some samples with different distances between grinding planes, it cannot be assumed that this has a distorting effect on the reconstruction in the 3D model because the section of the coarser grinding distance was not included in the model.

The values determined for the size of the cartilaginous ET can be compared with previous studies on this only to a limited extent. In a recent paper based on CBCT data of patients, an average length of the cartilaginous ET of 28.6 mm was provided but they also found that the ET becomes shorter in older patients [17]. Nevertheless, the shortest reported length of a cartilaginous ET in their study was 22.6 mm, which is approximately the average length in the current study. In earlier studies on histologic samples, smaller values (21.5–27 mm) for the length of the cartilaginous ET were provided [33], which fits better the measured lengths of 18.2 mm to 28.0 mm in the present study. Shrinkage of tissue due to embedding and fixation for histology was discussed as possible reason for these differences [17].

The shape of the stent could be elliptical in the images either due to squeezing the stent in the tissue or by cutting it not perpendicular to its axis. As the stents are not rigid tubes and the ET is not circular in shape, squeezing of the stents was expected. To avoid these uncertainties during evaluation of the stents, measurements were performed in a virtual plane perpendicular to the axis of the stent. However, using a virtual plane resulted in the determination of the stent areas not automatically by contrasting but by approximating it by an ellipse. This approximation was verified against the automatic measurement in the grinding plane. As in most cases the deviation was below 5%, this approach was considered suitable for the study.

The diameter of the ET is smallest in the isthmus region. Here, the height is about 3 mm and its width can be below 1 mm [34]. The ET widens towards the pharyngeal orifice to a maximum of about 8 mm, but is closed in its resting state. This shape influences the shape of the stents in the present study. The polymeric stent was one of the stents that was inserted through the isthmus. It was of a round shape at both ends but in the isthmus region, the long diameter was about twice the short one. The mentioned dimensions of the isthmus can explain these shape deviations. The same was observed for the metal stents. The cross-section area was larger on the pharyngeal side of the stents than on the side facing the isthmus, and only the stent that protrudes into the pharyngeal space exhibits a round shape again. The closer a stent comes to the isthmus, the more it is hindered in reaching its full size. Therefore, it can be assumed that, depending on the stent diameter, the isthmus region will provide an anatomical border for the stent in the direction of the middle ear and control of the insertion depth would be essential in clinical use. In addition, having a stent design with tapered cross-sections might be beneficial for application in the ET.

The large tubal cartilage appeared to be the main factor for the shape of the stent. The longest diameter of the stents was always approximately parallel to the long arm (medial lamina) of the cartilage. The only exception was stent NiTi 1a in the region where it was not unfolded. The medial part of the short diameter (D1) facing the medial lamina of the cartilage was always shorter than the lateral part (D2). Accordingly, the lateral half of the stents took a form closer to their original round shape. This indicates that the cartilage provides support for the stent. This was observed in fixed donor bodies. In living animals and patients, this might be different. Even though the tissue was still soft after fixation, fixation alters the properties of the tissue, and the turgor is not maintained.

Furthermore, a rotation of the long axis of the stent was observed. As the long axis of the stent was always approximately parallel to the tubal cartilage, this can also be interpreted as rotation of the tubal structures within the samples. The stent is round by production and only resistance from the outside can cause any deviations from a round shape. Some rotation in tubal structures was observed in sheep with a rotation of the tubal cartilage by 38° [32] without having a stent implanted. The values in the current study on human donors were generally smaller (between 6° and 32°), but these were only collected for the stented parts of the ET. So, a direct comparison was not advisable, but the torsion in the tube appears to be true also for humans. Only for stent NiTi 1a, the situation seemed to be different. This stent was not expanded in parts (compare Figure 3). This incomplete expansion might have induced the sudden change (swap) in long and short diameters, and has probably also caused the perceived negative values in overall rotation. Therefore, this stent cannot be compared to the other stents in all aspects of its behavior.

In addition to the anatomical form-giving structures, it was also expected to see differences due to the stent design. Therefore, areas and diameters at the same position in the ET were evaluated since comparable conditions with regard to the anatomical structures could be expected. Here, the area of the stent with open design (NiTi 2) was smallest compared to the other NiTi stents of the same nominal diameter. The relationship between long and small diameter was not distinguishable. Therefore, the smaller area cannot be explained by stronger squeezing of the stent. Less smooth changes in diameters over the entire length of the stent were observed for this stent compared to the other stents (compare Figure 6). This implies a visibly larger difference from plane to plane of undulating increasing diameter in the open design. With the open design, different parts of the stents were less connected. In these regions, the force the stent can put on the surrounding tissue might be diminished. This, in turn, might explain more pronounced changes in diameters along the stent. Therefore, the design of the stent also seems to have an effect on the behavior of the stent in the ET.

To develop a stent for application in the human ET, much more than these very first design considerations have to be regarded. The stent has to be reliably positioned in the intended region of the ET. Insertion tools have to be developed that enable reliable positioning. A patulous ET has to be avoided but the natural function of the ET shall be facilitated. To reach these goals, the best length, diameters, and shapes of the stent have to be investigated. It could also be that different patient etiologies or anatomies (e.g., long or short cartilaginous ET) require different stents. According to the first animal trials with coronary stents [14,15], tissue growth on top of the struts is to be expected and the mucosa seemed to close again. Therefore, late removal of a stent might cause greater damage but what about removal shortly after application in cases were the desired position was not achieved? There are many questions to answer, and the current manuscript can only be a first step in the development of a stent for application in the human ET.

## 5. Conclusions

The behavior of different stent prototypes made from Nitinol or polymeric materials was investigated after implantation into the ET of human body donors and compared to one coronary CoCr stent. NiTi stents seemed to adapt more to the shape of the ET than CoCr stents or polymeric stents. However, the larger diameter of the NiTi stents could have contributed to this observation. The closed design of NiTi stents appeared to have a more constant and less fluctuating course within the ET. All stents were able to open the lumen of the ET and a tapered design might be beneficial. Care has to be taken regarding insertion depth and therefore position of the stent in the ET. The tubal cartilage served as support for the stent. Further experiments to determine the correct size and positioning in order to support the ventilation of the middle ear without generating a patulous ET have to follow.

## Figures and Tables

**Table 1:** Overview of stents and their distribution.

	NiTi 1a	NiTi 1b	NiTi 2	NiTi 3a	NiTi 3b	CoCr	Polymer
Donor	22-018L	59-016R	47-017L	63-017L	14-017L	14-017R	59-016L
Sex	female	male	female	female	male	male	male
Age [years]	86	76	78	88	72	72	76
Type	Design 09 D5 L16 Identification No.: 5	Design 09 D5 L16 Identification No.: 3	Design 09ng D5 L17 Identification No.: 6	Design 12 D5 L16 Identification No.: 4	Design 12 D5 L16 Identification No.: 8	PRO-Kinetic Energy Identification No.: 08174960-11	IBMT 20 Identification No.: 229
Wall thickness	0.1 mm	0.1 mm	0.1 mm	0.1 mm	0.1 mm	0.06 mm	0.15 mm
Material	NiTi; Closed Design	NiTi; Closed Design	NiTi; Open Design	NiTi; Closed Design	NiTi; Closed Design	CoCr	PLLA-based blend
	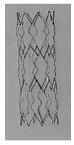	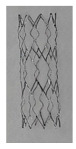	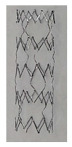	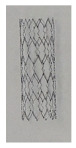	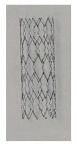	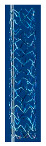 (expanded state)	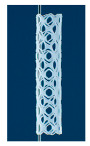 (expanded state)
Dimensions	15 mm × 5 mm	15 mm × 5 mm	15 mm × 5 mm	15 mm × 5 mm	15 mm × 5 mm	15 mm × 3 mm	20 mm × 3.5 mm

**Table 2:** Position of the stents in the ET.

	NiTi 1a, 22-018L	NiTi 2, 47-017L	NiTi 3a, 63-017L	NiTi 3b, 14-017L	CoCr, 14-017R	Polymer, 59-016L
Stent length as measured [mm]	13.1	15.8	15	15.7	13.2	19.9
Distance to Pharynx [mm]	6.2	−3	7.8	8.8	9.7	12
Distance to Isthmus [mm]	0.3	5.4	−1.7	3.5	−0.6	−6.8
Total length of cartilaginous ET [mm]	19.6	18.2	21.1	28	22.3	25.1

Negative values indicate that the stents go through the isthmus or protrude into the pharyngeal space. For visual orientation, please refer to Figure 1A.

**Table 3:** Cross-sectional area and diameters at both ends of the stents.

	NiTi 1a, 22-018L	NiTi 2, 47-017L	NiTi 3a, 63-017L	NiTi 3b, 14-017L	CoCr, 14-017R	Polymer, 59-016L
Area pharynx ostium [mm^2^]	9.8	13.8	10	15.1	6	3.2
Area isthmus [mm^2^]	1.5	4.7	1.9	7.1	3.8	2.9
Average area [mm^2^]	6	9.3	4.9	10	6.6	3.5
Diameter long Pharynx [mm]	4.4	4.4	5.6	5.3	3.9	2.2
Diameter short Pharynx [mm]	2.9	4	2.3	3.6	2	1.8
Diameter long Isthmus [mm]	1.9	4.1	3.1	5.3	3.3	2.1
Diameter short Isthmus [mm]	1	1.5	0.8	1.7	1.5	1.8
Average short diameter to tubal cartilage, D1 [mm]	0.9	1.3	0.5	0.9	0.9	0.7
Average short diameter away from tubal cartilage, D2 [mm]	1.1	1.5	0.9	1.4	1.4	1

D1 and D2 are referring to Figure 2.

**Table 4:** Stent areas and diameters at 6 mm distance from the isthmus.

	NiTi 1a, 22-018L	NiTi 2, 47-017L	NiTi 3a, 63-017L	NiTi 3b, 14-017L	CoCr, 14-017R	Polymer, 59-016L
Area [mm^2^]	5.7	5	5.7	7.7	7.1	4.0
Diameter short [mm]	2.2	1.5	1.7	1.8	2.7	1.9
Diameter long [mm]	3.3	4.1	4.2	5.5	3.4	2.6

## Data Availability

All the data that support the findings of this study are available on request from the corresponding author.

## References

[1] Di Martino E.F.N. (2013). Aktueller Stand der Tubenfunktionsdiagnostik Ein Update. HNO.

[2] Proctor B. (1967). Embryology and anatomy of eustachian tube. Arch. Otolaryngol..

[3] Schilder A.G.M., Bhutta M.F., Butler C.C., Holy C., Levine L.H., Kvaerner K.J., Norman G., Pennings R.J., Poe D., Silvola J.T. (2015). Eustachian tube dysfunction: Consensus statement on definition, types, clinical presentation and diagnosis. Clin. Otolaryngol..

[4] Poe D.S., Metson R.B., Kujawski O. (2003). Laser Eustachian Tuboplasty: A Preliminary Report. Laryngoscope.

[5] Schrom T. (2007). Treatment of chronic tube dysfunction. Use of the tube conductor. HNO.

[6] Politzer A. (1907). Geschichte der Ohrenheilkunde, Bd 1.

[7] Poe D.S., Grimmer J.F., Metson R. (2007). Laser Eustachian Tuboplasty: Two-Year Results. Laryngoscope.

[8] Sudhoff H., Schröder S., Reineke U., Lehmann M., Korbmacher D., Ebmeyer J. (2013). Therapy of chronic obstructive eustachian tube dysfunction: Evolution of applied therapies. HNO.

[9] Choi S.W., Lee S.H., Oh S.J., Kong S.K. (2020). Navigation-Assisted Balloon Eustachian Tuboplasty for Eustachian Tube Dilatory Dysfunction. Clin. Exp. Otorhinolaryngol..

[10] Ockermann T. (2009). Die Balloondilatation der Eustachischen Röhre zur Behandlung der obstruktiven Tubendysfunktion. Ph.D. Thesis.

[11] Llewellyn A., Norman G., Harden M., Coatesworth A., Kimberling D., Schilder A., McDaid C. (2014). Interventions for adult Eustachian tube dysfunction: A systematic review. Health Technol. Assess..

[12] Satmis M.C., van der Torn M. (2018). Balloon dilatation of the Eustachian tube in adult patients with chronic dilatory tube dysfunction: A retrospective cohort study. Eur. Arch. Otorhinolaryngol..

[13] Choi S.W., Oh S.J., Kim Y., Kwak M.Y., Suh M.W., Park M.K., Lee C.K., Park H.J., Kong S.K. (2021). A multicenter, randomized, active-controlled, clinical trial study to evaluate the efficacy and safety of navigation guided balloon Eustachian tuboplasty. Sci. Rep..

[14] Pohl F., Schuon R.A., Miller F., Kampmann A., Bültmann E., Hartmann C., Lenarz T., Paasche G. (2018). Stenting the Eustachian Tube to Treat Chronic Otitis Media—A Feasibility Study in Sheep. Head Face Med..

[15] Kang J.M., Kim S.H., Choi Y.J., Park Y., Ryu D.S., Kang W.S., Park J.H., Park H.J. (2022). Sirolimus-eluting cobalt-chrome alloy stent suppresses stent-induced tissue hyperplasia in a porcine Eustachian tube model. Sci. Rep..

[16] Ho A.C., Chan J.Y., Ng R.W., Ho W.K., Wei W.I. (2014). Stenting of the eustachian tube to prevent otitis media with effusion after maxillary swing approach nasopharyngectomy. Laryngoscope.

[17] Janzen-Senn I., Schuon R.A., Tavassol F., Lenarz T., Paasche G. (2020). Dimensions and positions of the Eustachian tube in Humans. PLoS ONE.

[18] Maleckis K., Anttila E., Aylward P., Poulson W., Desyatova A., MacTaggart J., Kamenskiy A. (2018). Nitinol Stents in the Femoropopliteal Artery: A Mechanical Perspective on Material, Design, and Performance. Ann. Biomed. Eng..

[19] Kang Y. (2019). A Review of Self-Expanding Esophageal Stents for the Palliation Therapy of Inoperable Esophageal Malignancies. Biomed. Res. Int..

[20] Snoeren R.M., Söderman M., Kroon J.N., Roijers R.B., de With P.H., Babic D. (2012). High-resolution 3D X-ray imaging of intracranial nitinol stents. Neuroradiology.

[21] Jensen L.O., Maeng M., Raungaard B., Kahlert J., Ellert J., Jakobsen L., Villadsen A.B., Veien K.T., Kristensen S.D., Ahlehoff O. (2020). Randomized Comparison of the Polymer-Free Biolimus-Coated BioFreedom Stent with the Ultrathin Strut Biodegradable Polymer Sirolimus-Eluting Orsiro Stent in an All-Comers Population Treated With Percutaneous Coronary Intervention: The SORT OUT IX Trial. Circulation.

[22] Wang R., Yuan Z., Li Q., Yang B., Zuo H. (2021). Damage evolution of biodegradable magnesium alloy stent based on configurational forces. J. Biomech..

[23] Jin L., Yao L., Yuan F., Dai G., Xue B. (2021). Evaluation of a novel biodegradable ureteral stent produced from polyurethane and magnesium alloys. J. Biomed. Mater Res. B Appl. Biomater..

[24] Pilgrim T., Heg D., Roffi M., Tüller D., Muller O., Vuilliomenet A., Cook S., Weilenmann D., Kaiser C., Jamshidi P. (2014). Ultrathin strut biodegradable polymer sirolimus-eluting stent versus durable polymer everolimus-eluting stent for percutaneous coronary revascularisation (BIOSCIENCE): A randomised, single-blind, non-inferiority trial. Lancet.

[25] Moravej M., Mantovani D. (2011). Biodegradable metals for cardiovascular stent application: Interests and new opportunities. Int. J. Mol. Sci..

[26] Fedorov A., Beichel R., Kalpathy-Cramer J., Finet J., Fillion-Robin J.-C., Pujol S., Bauer C., Jennings D., Fennessy F.M., Sonka M. (2012). 3D Slicer as an Image Computing Platform for the Quantitative Imaging Network. Magn. Reson. Imaging.

[27] Amatruda C.M., Bona Casas C., Keller B.K., Tahir H., Dubini G., Hoekstra A., Hose D.R., Lawford P., Migliavacca F., Narracott A.J. (2014). From histology and imaging data to models for in-stent restenosis. Int. J. Artif. Organs..

[28] Aguilar L.E., Tumurbaatar B., Ghavaminejad A., Park C.H., Kim C.S. (2017). Functionalized Non-vascular Nitinol Stent via Electropolymerized Polydopamine Thin Film Coating Loaded with Bortezomib Adjunct to Hyperthermia Therapy. Sci. Rep..

[29] Beranek J., Jaresova H., Rytz U. (2014). Use of nitinol self-expandable stents in 26 dogs with tracheal collapse. Schweiz. Arch. Tierheilkd..

[30] Kenry, Liu, B (2018). Recent Advances in Biodegradable Conducting Polymers and Their Biomedical Applications. Biomacromolecules.

[31] Malik N., Gunn J., Holt C.M., Shepherd L., Francis S.E., Newman C.M., Crossman D.C., Cumberland D.C. (1998). Intravascular stents: A new technique for tissue processing for histology, immunohistochemistry, and transmission electron microscopy. Heart.

[32] Schuon R., Schwarzensteiner J., Paasche G., Lenarz T., John S. (2021). Functional aspects of the Eustachian tube by means of 3D-modeling. PLoS ONE.

[33] Proctor B. (1973). Anatomy of the eustachian tube. Arch. Otolaryngol..

[34] Bezold F. (1882). Die Corrosions-Anatomie des Ohres.

